# Inhibitors of SARS-CoV-2 Main Protease (Mpro) as Anti-Coronavirus Agents

**DOI:** 10.3390/biom14070797

**Published:** 2024-07-04

**Authors:** Agnieszka Zagórska, Anna Czopek, Monika Fryc, Jakub Jończyk

**Affiliations:** Department of Medicinal Chemistry, Jagiellonian University Medical College, Medyczna 9, 30-688 Kraków, Poland; anna.czopek@uj.edu.pl (A.C.); monika.fryc@doctoral.uj.edu.pl (M.F.); jakub.jonczyk@uj.edu.pl (J.J.)

**Keywords:** COVID-19, SARS-CoV-2, main proteases (Mpro), 3-chymotrypsin-like protease (3CLpro), protein-ligand interactions, inhibitors, anti-coronavirus agents

## Abstract

The main protease (Mpro) of SARS-CoV-2 is an essential enzyme that plays a critical part in the virus’s life cycle, making it a significant target for developing antiviral drugs. The inhibition of SARS-CoV-2 Mpro has emerged as a promising approach for developing therapeutic agents to treat COVID-19. This review explores the structure of the Mpro protein and analyzes the progress made in understanding protein–ligand interactions of Mpro inhibitors. It focuses on binding kinetics, origin, and the chemical structure of these inhibitors. The review provides an in-depth analysis of recent clinical trials involving covalent and non-covalent inhibitors and emerging dual inhibitors targeting SARS-CoV-2 Mpro. By integrating findings from the literature and ongoing clinical trials, this review captures the current state of research into Mpro inhibitors, offering a comprehensive understanding of challenges and directions in their future development as anti-coronavirus agents. This information provides new insights and inspiration for medicinal chemists, paving the way for developing more effective Mpro inhibitors as novel COVID-19 therapies.

## 1. Introduction

The coronavirus disease 2019 (COVID-19) pandemic has left an irreversible mark on the world (over 18 million deaths) and destabilized the global economy, which still affects us today [1]. The rapid introduction of vaccination effectively reduces the risk of a severe disease course [2,3]. Combining nirmatrelvir with ritonavir effectively reduces the risk of hospitalization or death in high-risk COVID-19 patients who are prone to severe illness [4,5,6]. The insights gained from COVID-19 and other infectious diseases concerning drug repurposing strategies, pan-coronavirus drug targets, in vitro assays, animal models, and platform trial design are crucial for developing therapeutics to combat COVID-19, long COVID, and future pathogenic coronavirus outbreaks [7,8,9]. However, the threat has not been eliminated.

Coronavirus groups are characterized by a relatively high mutation rate, e.g., a spontaneous mutation rate of 1.3 × 10^−6^ for SARS-CoV-2) [10,11,12]. Mutation leads to the formation of strains with greater virulence, that are resistant to immunization caused by vaccinations, or are characterized by a more severe course of the disease. Moreover, some studies showed that several mutational pathways confer resistance to nirmatrelvir [13]. Mutation has been observed to alter the structure of the protease (e.g., the binding pocket and conformation) and reduce the binding affinity of nirmatrelvir or ensitrelvir, reducing drug efficacy. The polymorphic nature of Mpro, which can undergo various mutations, promotes this situation, and the exact impact of each mutation can depend on the specific viral strain. Moreover, a combination of mutations can confer a higher level of resistance than a single mutation. Understanding these mutations is crucial for developing and optimizing antiviral therapies targeting SARS-CoV-2 and other coronaviruses [14,15,16,17]. Consequently, there is a pressing need for more targeted pharmacological treatments to complement vaccination efforts in preventing and treating COVID-19.

The replication cycle of SARS-CoV-2 involves several stages, beginning with viral entry into host cells, followed by genome replication, protein synthesis, assembly of new viral particles, and finally release from the host cell to infect other cells. The SARS-CoV-2 genome encodes two polyproteins, pp1a and pp1ab, along with four structural proteins. These polyproteins undergo cleavage by the essential SARS-CoV-2 main protease (Mpro or 3CL protease) at 11 specific sites, resulting in shorter, nonstructural proteins (nsps) crucial for viral replication, mainly orchestrating the synthesis of subgenomic RNA, crucial for the host cell’s production of viral structural proteins such as the envelope, membrane, spike, and nucleocapsid. Additionally, nsps are crucial in suppressing the antiviral response activated by innate immunity or interferons [18].

Mpro’s essential function and unique structure make it an attractive target for developing specific antiviral drugs. By inhibiting Mpro activity, it is possible to disrupt viral replication, promote an immune response, and potentially halt the progression of infection.

Moreover, one of the primary advantages of targeting Mpro lies in its conservation among coronaviruses. Despite the genetic diversity observed within the coronavirus family, Mpro exhibits high sequence and structural similarity across different strains. This conservation facilitates the development of broad-spectrum antiviral drugs effective against various coronaviruses, including SARS-CoV-2, Middle East respiratory syndrome coronavirus (MERS-CoV), and potentially future emerging coronaviruses. Given the lack of a human homolog for Mpro and its distinct substrate specificity that does not align with any known human protease, there is an opportunity to design Mpro inhibitors that are effective and precise, with minimal inhibitory effects on human proteases. This would result in a decrease in the side effects typically associated with many antiviral agents.

This review aims to collect knowledge regarding the structural features and inhibitory mechanisms of SARS-CoV-2 Mpro inhibitors to offer insights and inspiration to medicinal chemists for designing and developing more efficacious Mpro inhibitors as novel anti-coronavirus agents.

## 2. The Structure of Mpro 

**Figure 1:**
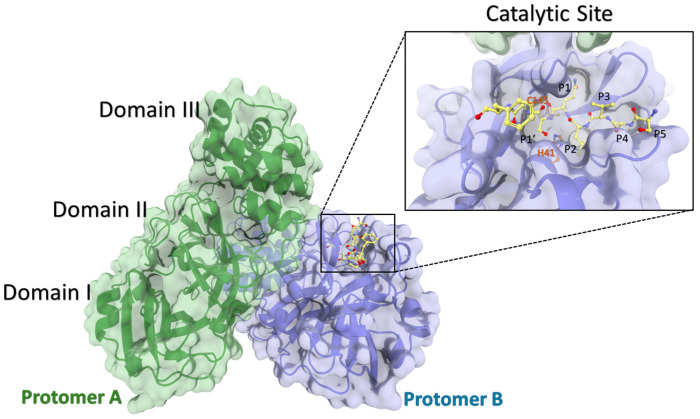
Arrangement of the Mpro homodimer (built utilizing PDB complex: 7MGS [21]) and a comprehensive illustration of the viral N-terminal autoprocessing substrate nsp4/5 (depicted in yellow) while it is bound to its catalytic site, highlighting the catalytic residues His41 and Cys145 in orange.

Structurally, SARS-CoV-2 Mpro is a homodimer composed of two protomers labeled A and B (Figure 1). Each protomer comprises three domains: domains I (residues 8–101) and II (residues 102–184) consist of antiparallel β-barrels, collectively forming a chymotrypsin-like structure; domain III (residues 201–306), primarily composed of α-helices, governs the catalytic process. Mpro is characterized as a three-domain cysteine protease with a catalytic dyad composed of Cys145–His41 situated in the cleft between domains I and II (P1 region) [19,20]. The histidine (His41) forms a hydrogen bond with a water molecule, which also interacts with the side chains of aspartate (Asp187) and histidine (His164). Asp187 is further stabilized by a salt bridge with a nearby arginine (Arg40). This arrangement allows His41 to function as a base, extracting a proton from the catalytic cysteine (Cys145) side chain, thereby activating it for the nucleophilic attack that cleaves the polypeptide. The interaction between the N-terminus of domains I + II and the C-terminus of domain III leads to the formation of a reversible dimer in Mpro, which is further stabilized in the presence of substrate. 

### 2.1. Substrate Recognition

Mpro recognizes and cleaves the amino acid sequence of the substrate with specificity, mostly at Leu-Gln (Ser, Ala, or Gly) sequences, while no known human proteases have a similar specificity. 

**Figure 2:**
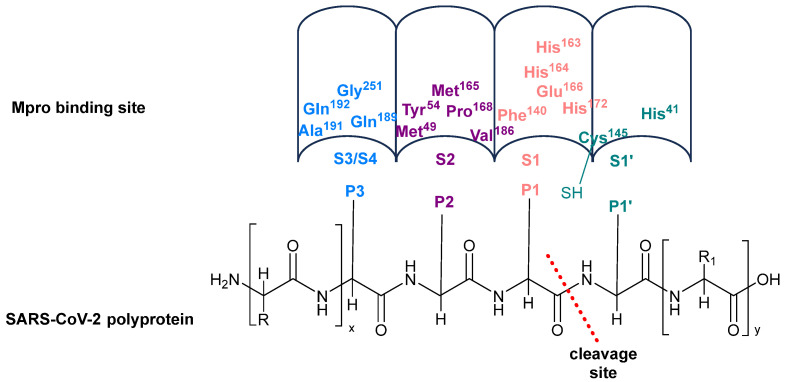
SARS-CoV-2 Mpro binding site with viral polyproteins. Mpro splits the viral polyproteins (pp1a and pp1ab) at 11 sites. The cleavage involves a characteristic motif: Leu-Gln (Ser, Ala, Gly). Variability exists in the amino acid sequence at these cleavage sites, with glutamine consistently found at P1 and a preference for leucine at P2. Hydrophilic and hydrophobic residues can be inserted at the P3 position.

In addition to the catalytic pair composed of His41 and Cys145, Mpro hosts a water molecule within its active site, displacing the typical aspartate residue found in the conventional catalytic triad of other proteases, usually associated with histidine and cysteine [22]. In the process of peptide bond hydrolysis, His41 initiates activation of the nucleophilic -SH group of Cys145 by deprotonation, followed by stabilization of the resultant adduct through the formation of the “oxyanion hole,” which is constituted by the backbones of Gly143 and Cys145. The S1 region, delineated by the side chains of Phe140, His163, His164, Glu166, and His172, exhibits high specificity towards glutamine residues. Region S2 comprises hydrophobic amino acids, including Met49, Tyr54, Met165, Pro168, and Val186, while regions S3/S4, which are notably exposed to the solvent, encompass residues such as Gln189, Ala191, Gln192, and Gly251 (Figure 2) [23]. 

### 2.2. The Catalytic Mechanism for the Cleavage of Polyproteins by Mpro

**Figure 3:**
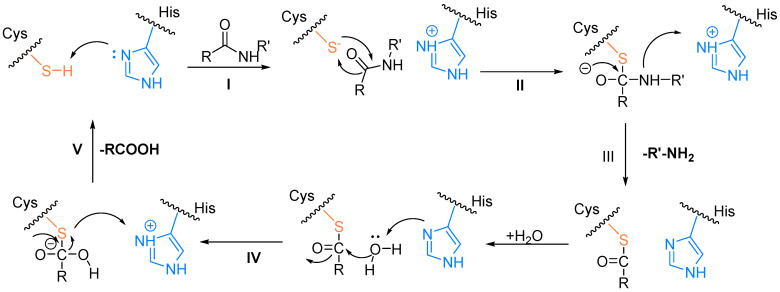
The catalytic mechanism of hydrolysis by Mpro.

The catalytic mechanism of Mpro is, like that of other cysteine proteases, based on the nucleophilic residue, which can participate in the reaction mechanism (Figure 3). Initially, the -SH group of Cys145 from the catalytic dyad undergoes deprotonation facilitated by the nearby His41 (step I). It has been proposed that the zwitterionic state of the Cys145–His41 dyad, rather than its neutral form, initiates the reaction in step I. Subsequently, the resulting anionic sulfur attacks the carbonyl carbon of the scissile amide bond (step II). This action releases a peptide product with an amine terminus, while the His41 returns to its deprotonated state (step III). Finally, the produced thioester undergoes hydrolysis, liberating the carboxylic acid (step IV), thus restoring the catalytic dyad to its initial state, primed for the subsequent proteolytic cycle (step V) [24,25,26]. 

## 3. Classification of Mpro Inhibitors

Mpro inhibitors can be classified based on their binding kinetics, origin, and chemical structure. Here is a brief overview of each classification.

### 3.1. Classification of Mpro Inhibitors by Binding Kinetics 

**Figure 4:**
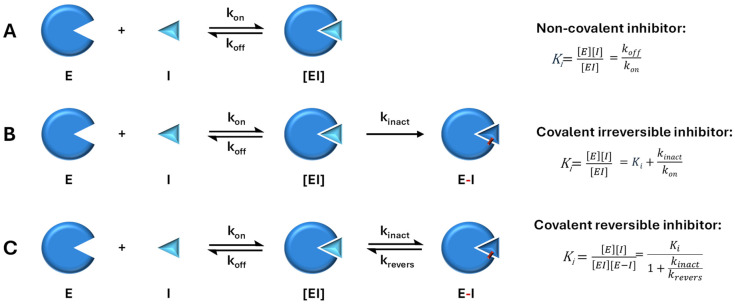
Comparison of non-covalent and covalent interactions between an enzyme and an inhibitor. (**A**) Interaction of a non-covalent reversible inhibitor with an enzyme. Interaction of covalent, irreversible (**B**), and reversible (**C**) inhibitors with an enzyme.

Mpro inhibitors exhibit binding to the protease through non-covalent (A) and covalent irreversible (B) or reversible (C) mechanisms. A schematic of the mode of action of Mpro inhibitors and kinetics parameters is presented in Figure 4.

#### 3.1.1. Non-Covalent Inhibitors of Mpro

**Figure 5:**
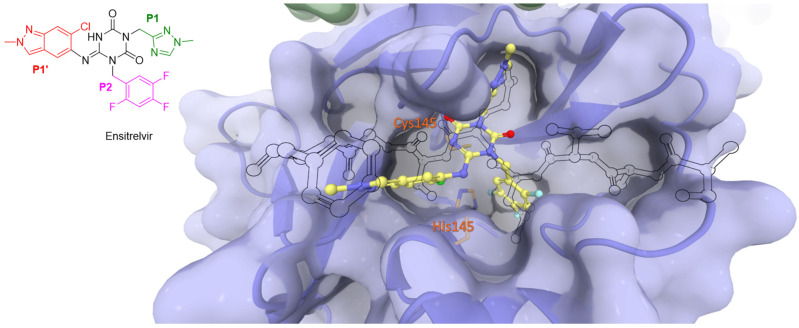
The binding mode of ensitrelvir (depicted in yellow) with the catalytic site of SARS-CoV-2 Mpro compared to the viral N-terminal autoprocessing substrate nsp4/5 (as a black outline). Catalytic dyad residues His41 and Cys145 are highlighted in orange.

Non-covalent inhibitors bind reversibly to the enzyme, forming an enzyme–inhibitor complex. Non-covalent means that the inhibitor can bind to and dissociate from the enzyme multiple times. The equilibrium constant (*K*_i_) is then defined as the ratio of the unbinding and binding rate constants (*k*_off_/*k*_on_). Non-covalent inhibitors competitively bind to the catalytic site through crucial hydrogen bonds and non-polar interactions. This binding can cause distortions in the sub-pockets and displace essential water molecules, ultimately blocking access to the catalytic site. An example of this mechanism is ensitrelvir, approved in Singapore in November 2023, as shown in Figure 5 [27]. 

**Figure 6:**
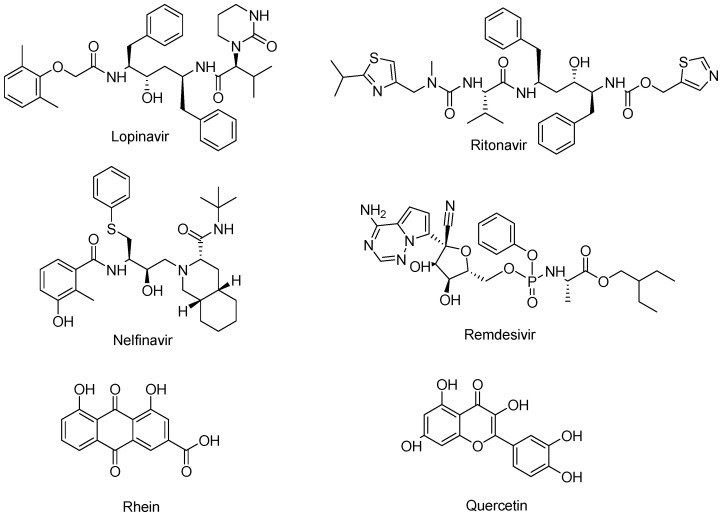
Examples of non-covalent inhibitors of Mpro.

Most of the potential non-covalent inhibitors of Mpro have been repurposed from HIV protease inhibitors (lopinavir/ritonavir/nelfinavir, Figure 6) [28]. Moreover, remdesivir, rhein, quercetin, and ensitrelvir (approved in Singapore in November 2023) showed inhibitory effects against Mpro through non-covalent interactions.

#### 3.1.2. Covalent Inhibitors of Mpro

Overall, covalent inhibitors interact with Mpro in two steps. The first stage involves the reversible interaction between the enzyme and inhibitor, where the amino acids in the binding pocket interact with the peptide backbone. Next, the nucleophilic attack of the thiol group on a carbon atom of the inhibitor results in the formation of a new bond. The inhibition can be either reversible or irreversible, depending on the strength of this newly formed covalent bond [29,30]. Covalent inhibitors are designed by incorporating the electrophilic group into peptidomimetic inhibitors already known to bind non-covalently with micromolar affinity [31].

Irreversible covalent inhibitors form a covalent bond with the target enzyme, leading to permanent inhibition of enzyme activity. Therefore, the equilibrium constant (*K*_i_) is not directly applicable in the case of irreversible covalent inhibitors. Instead, in the context of irreversible covalent inhibition, another parameter known as the “inactivation constant” (*K_i_*) or “*K*_inact_/*K_on._* ratio” is a second-order rate constant, often used to describe the potency of the inhibitor. *K_i_* is essentially the concentration of inhibitor at which half of the enzyme molecules are irreversibly inhibited. A higher ratio indicates greater potency of the inhibitor because it reflects a higher rate of enzyme inactivation relative to the concentration required for inhibition. A compound named N3, a peptidomimetic Michael acceptor inhibitor, was one of the first inhibitors that proved its effectiveness in inhibiting Mpro from multiple coronaviruses, including SARS-CoV, MERS-CoV, and SARS-CoV-2 [32]. Its structure largely resembles the natural enzyme substrate. 

**Figure 7:**
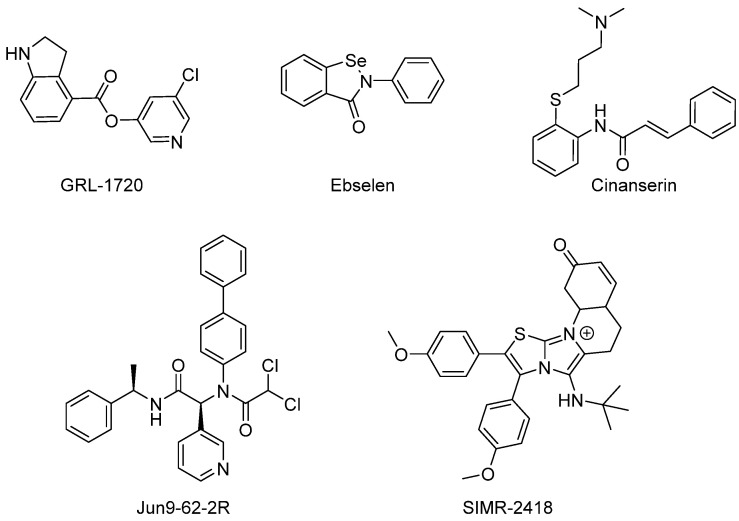
Examples of irreversible covalent inhibitors of Mpro.

Alongside the N3 inhibitor, many other inhibitors such as GRL-1720 [33], cinanserin [32], Jun9-62-2R [26], SIMR-2418 [34], and ebselen [32] have demonstrated potent inhibition of Mpro through irreversible covalent binding to the catalytic cysteine residue (Figure 7). 

**Figure 8:**
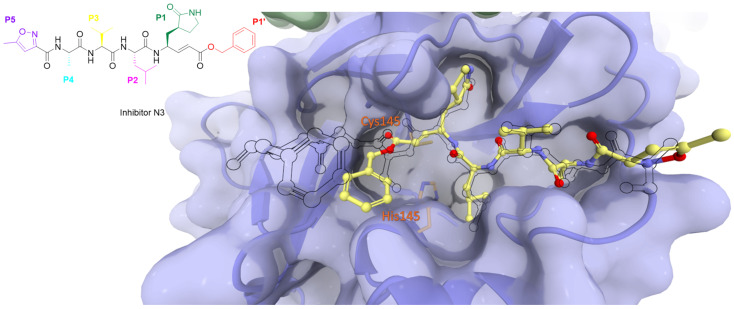
The binding mode of inhibitor N3 (depicted in yellow) with the catalytic site of SARS-CoV-2 Mpro compared to the viral N-terminal autoprocessing substrate nsp4/5 (as a black outline). Catalytic dyad residues His41 and Cys145 are highlighted in orange.

The catalytic sulfur of Cys145 is covalently bound to an electrophilic moiety termed P1′, acting as a reactive warhead that mimics the amide peptide found in viral polyproteins. For successful covalent inhibition, inhibitors must satisfy fundamental structural criteria: a P1 segment, typically a cyclic glutamine analogue, capable of engaging in hydrogen bonds and hydrophobic interactions with amino acids like His163, Glu166, and His172; and P2 and P3 moieties (see N3, Figure 8), extending into hydrophobic pockets (S2, S3, and S4) and featuring substituent groups conducive to favorable hydrophobic interactions with the amino acids residing in these regions.

**Figure 9:**
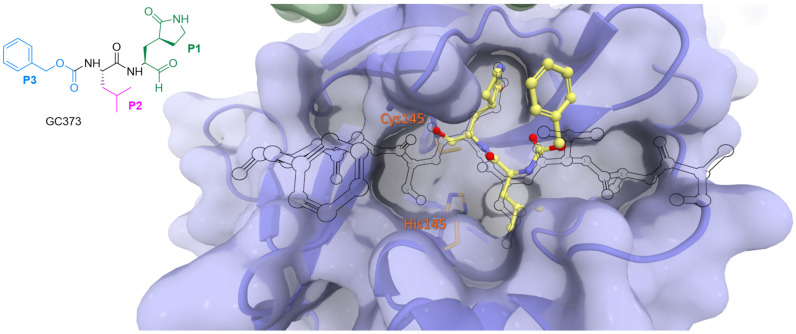
The binding mode of GC-373 (depicted in yellow) with the catalytic site of SARS-CoV-2 Mpro compared to the viral N-terminal autoprocessing substrate nsp4/5 (as a black outline). Catalytic dyad residues His41 and Cys145 are highlighted in orange.

Reversible covalent inhibitors form a covalent bond with the target enzyme, but this bond can be broken under certain conditions, allowing the enzyme to regain its activity. In reversible covalent inhibition, the equilibrium constant (*K*_i_) represents the dissociation constant of the enzyme–inhibitor complex. It quantifies the inhibitor’s affinity for the enzyme and is defined as the ratio of the rate constants for dissociation (*k*_revers_) and association (*k*_inact_) of the enzyme–inhibitor complex, similar to non-covalent inhibitors. Aldehyde-based reversible covalent inhibitor GC-373 and its prodrug GC-376 were initially designed as inhibitors of feline coronavirus (FCoV) Mpro but proved to bind effectively to SARS-CoV-2 Mpro [35]. The binding of GC-373 to the catalytic center of Mpro is presented in Figure 9. 

**Figure 10:**
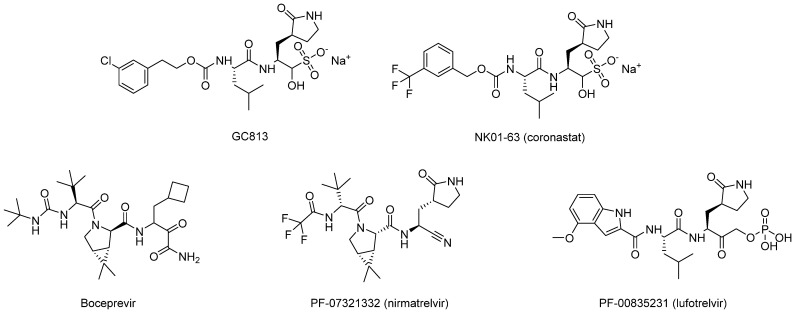
Structure of selected reversible SARS-CoV-2 Mpro covalent inhibitors.

Other examples of covalent Mpro inhibitors with reversible effects include GC-813, NK01-63 (coronastat), boceprevir, PF-07304814 (lufotrelvir), and PF-07321332 (nirmatrelvir) (Figure 10) [36,37]. 

The covalent inhibitors PF-07304814 and GC376 showed promising inhibition of SARS-CoV-2 in vitro but displayed poor oral bioavailability in rats (1.4% and 3%, respectively) [38]. These first-generation Mpro inhibitors established starting points for the optimization process. A cyclized pyrrolidone was designed to mimic the glutamine side chain to prevent the formation of a cyclized product with the warhead. Next, incorporating a 6,6-dimethyl-3-azabicyclo [3.1.0]hexane at the P2 position eliminated the hydrogen bond donor within the P2/P3 amide linkage. Replacing the hydrogen bond donor of the α-hydroxymethyl ketone covalent warhead with a nitrile group compound improved oral bioavailability.

### 3.2. Classification of Mpro Inhibitors by Origin

Natural Products: These inhibitors are derived from natural sources, such as plants, marine organisms, or microorganisms. Due to their diverse chemical structures and biological activities, natural products often serve as lead compounds for drug discovery. Krüger et al. [39] assessed 31 natural compounds and their derivatives for their potential as inhibitors of SARS-CoV-2 Mpro. Robinetin and rosmarinic acid, along with its derivative salvianolic acid A, were identified as novel ligands for Mpro, while the inhibitory effect on Mpro was further confirmed for myricetin, oridonin, scutellarein, and L-epigallocatechin gallate. The remarkable antiviral potency that was observed, particularly with robinetin displaying an EC50 of 1.3 nM, underscores these compounds’ potential for further investigation as antiviral agents against COVID-19. An integrated metabolomic and proteomic approach for screening natural extracts, particularly those containing compounds with catechol or pyrogallol moieties, identified SARS-CoV-2 Mpro inhibitors from crude natural extracts [40]. In their study, Zhang et al. [41] tested 60 herbs for possible antiviral properties. Lonicera japonica Flos displayed the highest level of effectiveness against Mpro, with an IC50 value of 37.82 μg/mL. Gallic acid and quercetin extracted from this source showed inhibition of Mpro, with IC50 values below 10 µM.

**Figure 11:**
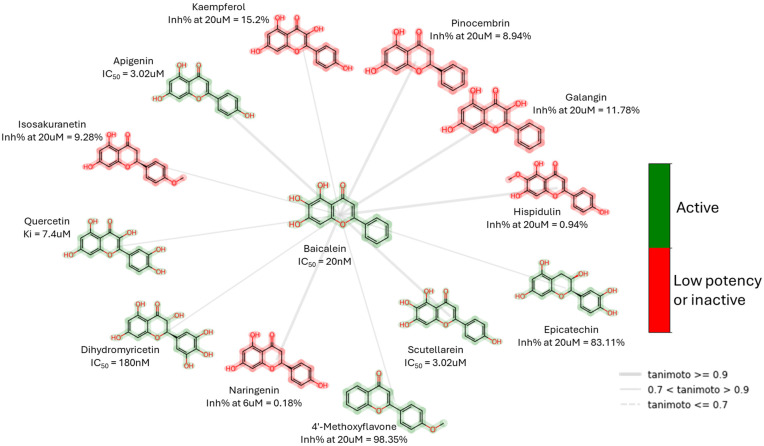
Baicalein, a non-covalent SARS-CoV-2 Mpro inhibitor, and examples of its related compounds (maximum common substructure (MCS)) with known Mpro inhibitory activity. The low potency criterion was IC_50_ or *K*_i_ > 10 μM or Inh% < 75%.

In order to identify potential drug candidates against SARS-CoV-2 Mpro in natural products, computer-based techniques have been extensively employed. These methods, including machine learning, molecular docking, and other virtual screening techniques, enable the identification of potential inhibitors. Flavone derivatives such as Baicalein (Figure 11) or labdane diterpenoid andrographolide were identified with molecular docking and molecular dynamics protocols as Mpro inhibitors with anti-SARS-CoV-2 activity [42]. Through structure-based pharmacophore screening and QSAR analysis of natural product repositories, novel chemical scaffolds for Mpro inhibitors were discovered. Among these, pseurotin A, lactupicrin, and alpinetin exhibited IC50 values in the micromolar range [43].

Machine learning-based virtual screening was performed on the library of 4000 phytochemicals, leading to the identification of 26 potential inhibitors of the Mpro [44]. These hits were then docked. By employing virtual screening techniques such as molecular docking and molecular dynamics simulation, researchers successfully identified three promising phytochemicals (cyanidin 3-O-galactoside, β-carotene, and epicatechin) from cranberry as potential inhibitors of SARS-CoV-2 Mpro. Among them, cyanidin 3-O-galactoside exhibited the most potent inhibitory potential, with an IC50 value of 9.98 μM [45].

Flavone derivatives such as baicalein (Figure 11) or labdane diterpenoid andrographolide were already described as Mpro inhibitors with anti-SARS-CoV-2 activity.

Repurposed Drugs: Discovering medications for newly identified diseases is increasingly challenging today. A recent approach to address this challenge is drug repurposing, which pertains to utilizing existing medications for novel therapeutic purposes. The advantage of utilizing compounds from drug-repurposing libraries is that these molecules already have proven cell permeability and bioactivity. Unfortunately, systematic, hypothesis-free, large-scale drug library screening has yet to yield effective disease treatments [46].

**Figure 12:**
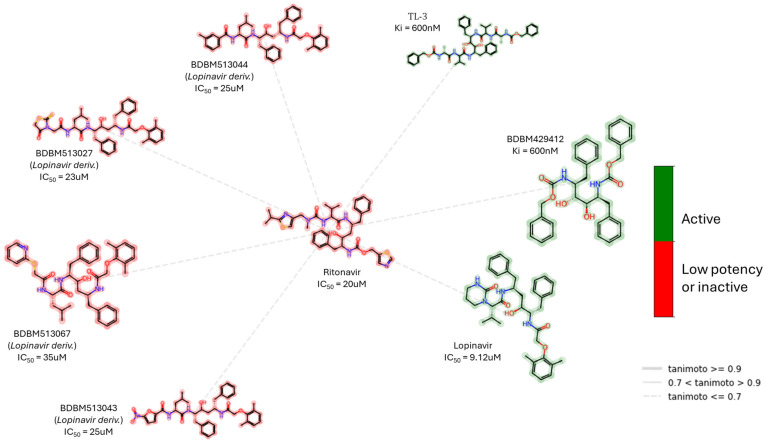
Ritonavir, a non-covalent inhibitor of SARS-CoV-2 Mpro, and examples of its structurally closely related compounds (maximum common substructure (MCS)) with known Mpro inhibitory activity from the PubChem Database. The low potency criterion was IC_50_ or *K*_i_ > 10 μM.

Once again, computer techniques played a crucial role in the initial stages of evaluating repositioned compounds. Structure-based virtual screening, which combines molecular docking and molecular dynamics techniques, has frequently identified potential candidates for COVID-19 treatment. Ambrosio et al. [47] used a molecular docking technique to discover potential repurposed antiviral compounds (seven cephalosporins and the oral anticoagulant betrixaban) against the SARS-CoV-2 Mpro. Cefadroxil and cefoperazone demonstrate moderate activity, with IC50 values of 2.4 µM and 4.9 µM, respectively. Betrixaban shows promising activity, with an IC50 value of 0.9 µM. Existing antiviral drugs from the group of HIV protease inhibitors, lopinavir, ritonavir, and nelfinavir, were classified as non-covalent inhibitors of Mpro. Lopinavir, ritonavir, and their selected structural analogues are presented in Figure 12. This discovery contributed to the further exploration of HIV protease inhibitors through pharmacophore modelling and covalent-docking-based screening, including C-2 symmetric peptidomimetics. TL-3 and similar molecules were found to have potent inhibitory effects on Mpro, reaching the nanomolar concentration range [48]. Sorafenib, identified as a potential Mpro [49] inhibitor through a comparable virtual screening protocol on VEGFR-2 inhibitors, exhibited an inhibitory ability of 25% at a concentration of 20 µM in subsequent studies (ChEMBL AssayID: CHEMBL4495564). The application of molecular docking and molecular dynamics techniques suggested that minocycline and rifampicin, antimycobacterial drugs, have promising potential as inhibitors of Mpro. In vitro tests revealed disappointing results for minocycline, showing a weak activity level with an IC50 of 5 mM [50].

Synthetic Compounds: These inhibitors are chemically synthesized in the laboratory and optimized for specific interactions with Mpro based on structure–activity relationship studies and medicinal chemistry approaches.

**Figure 13:**
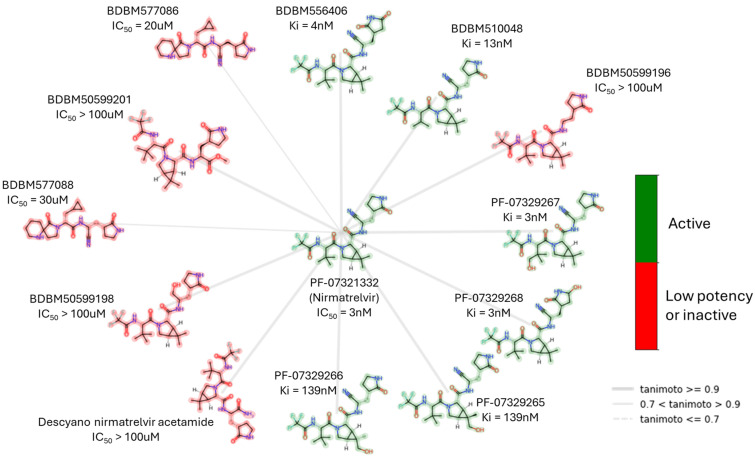
Nirmatrelvir, a covalent, reversible SARS-CoV-2 Mpro inhibitor, and examples of its structurally closely related compounds (maximum common substructure (MCS)) with known Mpro inhibitory activity from the PubChem Database. The low potency criterion was IC_50_ or *K*_i_ > 10 μM or Inh% at 20 μM < 75%.

Recent drug discovery pipelines focus on covalent SARS-CoV-2 Mpro inhibitors since this was a productive line of research during the outbreak. Identifying effective non-covalent Mpro inhibitors came later but was considered more challenging. Researchers utilized diverse strategies to discover new Mpro inhibitors, such as high-throughput screening [51,52,53,54,55,56], target-based rational drug design, and fragment-based screening and optimization [32,57,58,59,60,61,62,63,64,65]. An example of such meticulous exploration is the search for nirmatrelvir analogues, some of which are illustrated in Figure 13. There has been extensive research on the impact of substituting the nitrile with other groups that can form a covalent bond with cysteine and modifying the inhibitor to have a non-covalent nature. In the second scenario, most of the derived compounds did not demonstrate capacity to block Mpro. By analyzing nirmatrelvir metabolites, numerous compounds were identified that maintained strong activity, even with hydroxyl groups in the P2 and P3 regions. Computer methods have significantly supported many campaigns to find new Mpro inhibitors.

One of the most popular approaches was searching extensive chemical compound databases for new chemotypes. A combination of classic virtual screening techniques (pharmacophores, molecular docking, and molecular dynamics) and modern approaches like machine learning or hybrid methods were utilized to achieve this goal. In their study, Lee et al. employed various methods, including 3D shape and electrostatic similarity, molecular docking, and fingerprint similarity, to perform a virtual screening of 500,282 compounds from a Korean compound bank [61]. To identify promising Mpro inhibitor candidates, Luo et al. combined the Partial Least Square (PLS) QSAR model with molecular docking and molecular dynamics techniques. They focused on designing new derivatives of 6,6-dimethyl-3-azabicyclo[3.1.0]hexane [62]. Using molecular docking, Zhai et al. conducted a virtual screening of InterBioScreen’s compound database, which contains more than 550,000 high-quality compounds. The activity of the compounds was assessed using the QSAR model [63]. Two compounds out of the 71 potential inhibitors selected in virtual screening exhibited moderate inhibitory activity, with IC50 values of 19 µM and 38 µM.

Using computer techniques often leads to identifying hit structures, which then serve as a starting point for optimizing and designing more effective molecules. Mercorelli et al. conducted an initial virtual screening of approximately 800,000 compounds from Specs and ChemDiv vendors using a hybrid protocol that combined ligand-based and target-structure-based approaches [64]. Among the 30 initial virtual screening hits, seven have shown inhibitory activity against Mpro, with IC50 values ranging from 26.6 µM to 141.5 µM. By utilizing information about the potential binding mode of ligands in Mpro’s active site, further optimization resulted in even more potent inhibitors, with the most active one having an IC50 value of 17.1 µM. Subsequent experimental verification has not been conducted for many of the published results from virtual screening.

The task of evaluating the biological potential of the identified compounds from two large molecular docking screens was undertaken by Rossetti et al. [65]. Among the 486 hits that were tested, five compounds showed inhibition of Mpro at a level exceeding 25% at a concentration of 40 µM. Employing a systematic approach to find analogues of the selected chemotypes (such as diamino-quinazoline and dihydro-quinolinone), researchers successfully uncovered novel Mpro inhibitors exhibiting IC50 values spanning from 1 μM to 24 μM.

The research community has acknowledged and harnessed the effectiveness of machine learning techniques and neural network models in the search for new Mpro inhibitors. These approaches have been commonly used with molecular modeling techniques in numerous protocols. Li et al. aimed to use the MproI-GEN, a deep learning model composed of long short-term memory modules, for the de novo design of Mpro inhibitors [66]. Joshi et al. combined the Automated Modeling Engine (AME) for automated covalent docking of specified electrophilic warheads with a 3D scaffold, a neural network-based generative model tool they had previously developed [67]. This approach created a collection of potential Mpro inhibitors, and their ability to strongly bind to the enzyme was evaluated through comprehensive all-atom MD simulations. In experimental studies, three chloroacetamide warhead-based hits have demonstrated their effectiveness as promising ligands, with Ki values of 11.5, 5.97, and 5.27 μM.

In their research, Gentile et al. attempted to explore a vast collection of molecules. This collection comprised over 40 million compounds formed by combining libraries of purchasable chemicals from ZINC15 and enumerated Enamine REAL Space [68]. To address this challenge, they devised a classification model using neural networks trained with chemical fingerprints and docking scores [69]. The objective was to eliminate low-scoring molecules before their docking with Autodock GPU, Glide SP, FRED, ICM, and QuickVina2, along with pharmacophore modeling tools. Out of the compounds chosen through consensus protocols, 1283 were subjected to testing in an Mpro enzymatic assay. Among them, 117 compounds exhibited an inhibition of SARS-CoV-2 Mpro enzymatic activity greater than 10% at a concentration of 5 μM.

### 3.3. Classification of Mpro Inhibitors by the Chemical Structure

Two prominent classes of Mpro inhibitors are peptidomimetics and small molecules. While both have distinct advantages and challenges (Table 1), the transition from peptidomimetics to small-molecule inhibitors represents a significant evolution in the drug design landscape.

Peptidomimetics were developed to transform peptides into more drug-like molecules [70,71,72]. By definition, peptidomimetics are synthetic compounds designed to mimic the binding ability of a natural substrate to its target site without undergoing the enzyme-catalyzed reaction [73,74,75]. These molecules offer higher metabolic stability, improved bioavailability, and enhanced receptor affinity and selectivity than peptides [74,75]. However, their in vivo activity often falls short due to low bioavailability from poor oral absorption and high metabolic susceptibility. Newer generations of peptidomimetics are designed to be less peptide-like, incorporating non-natural amino acids or structural elements that avoid peptide bonds. These modifications can enhance in vivo activity but may increase synthesis costs. While peptidomimetics offer high specificity and stability, their complex synthesis and limited bioavailability pose challenges.

The transition from peptidomimetics to small molecules is a complex process that requires a deep understanding of both peptide chemistry and small-molecule drug design. Firstly, structure–activity relationship (SAR) analysis determines the functional groups in the peptidomimetic, which are crucial for binding to the target and biological activity. Structure optimization focuses on the core scaffold responsible for the activity, removing non-essential molecule parts to reduce molecular weight and complexity to comply with Lipinski’s Rule of Five. Designing effective small-molecule Mpro inhibitors involves leveraging various strategies to enhance binding affinity and selectivity while maintaining favorable pharmacokinetic properties. One approach is using covalently connected building blocks to prepare many compounds. This strategy has proven successful in the design of SARS-CoV-2 Mpro inhibitors, where different covalent warheads and modifications at key positions (P1, P2, and P3) have been explored.

Peptidomimetic and small-molecule-based inhibitors can bind to Mpro through non-covalent and covalent links. Electrophilic warheads can “trap” the thiol group of Cys145 through an irreversible or reversible covalent bond, thus inactivating the enzyme. The prevailing peptide-based covalent inhibitors bear a peptidic scaffold with highly active warheads.

#### 3.3.1. Peptidomimetics

The starting point for the design of Mpro inhibitors was mimicking peptide substrates. For example, the peptidyl chloromethyl ketone (CMK) substrate analogue was derived from the residues of the N-terminal auto-cleavage site of transmissible gastroenteritis virus (TGEV) Mpro [76,77]. Peptidomimetics are synthetic constructs designed to replicate the natural structure of peptides or proteins. They interact with biological targets like the original substrate and exhibit enhancements over natural peptides, such as increased metabolic stability, improved pharmacokinetic properties, heightened potency, and more.

**Figure 14:**
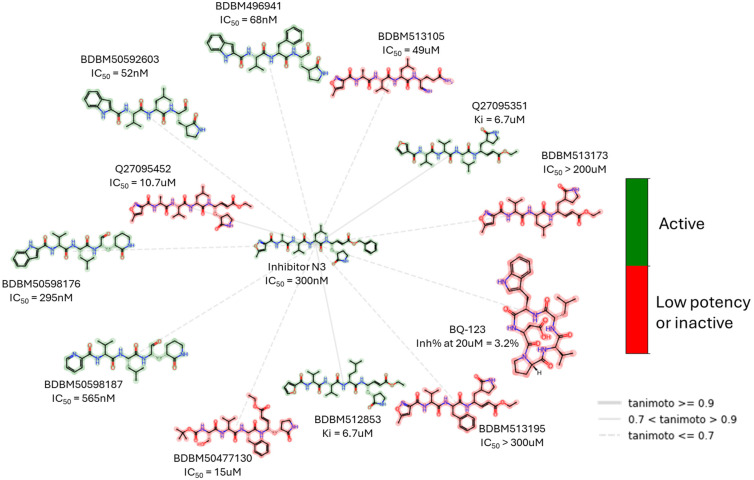
Peptidomimetic covalent SARS-CoV-2 Mpro inhibitor N3 and examples of its structurally closely related compounds (maximum common substructure (MCS)) with known Mpro inhibitory activity from the PubChem Database. The low potency criterion was IC_50_ or *K*_i_ > 10 μM or Inh% at 20 M < 75%.

Analysis of the binding modes of peptidomimetic Mpro inhibitors revealed shared characteristics among them [27,78]. The typical inhibitor structure can be delineated into four segments (P1′, P1, P2, P3). P1 to P3 constitute the peptide backbone, which may incorporate modified amino acids to bolster peptidomimetic attributes and furnish the specific recognition motif for SARS-CoV-2 Mpro. Moreover, utilizing knowledge of Mpro substrate specificity, there is a Gln residue at P1, while at P2, large hydrophobic residues are typically favored (e.g., aromatic rings capable of engaging in π–π stacking interactions). Conversely, the P1′ subsite generally accommodates small hydrophobic residues. Thus, for peptidomimetic Mpro inhibitors, an electrophilic moiety at the C-terminus (warhead) occupies the P1′ site, essential for the protease’s covalent inactivation (Figure 14). These C-terminal electrophilic warheads are categorized based on the various functional groups present at P1′ as peptidyl chloromethyl ketones, aza-peptide epoxides, peptides with a Michael acceptor, an aldehyde, a phthalhydrazido group, a nitrile group, an aldehyde bisulfite adduct, and an α-ketoamide [79,80,81,82,83,84,85].

#### 3.3.2. Small Molecules

From a chemical point of view, most of the compounds exhibited a dipeptidomimetic or tripeptidomimetic structure with carbonyl, α-ketoamide, Michael acceptor, α-haloacetamide, and nitrile warhead. However, some small-molecule Mpro inhibitors, such as activated ester derivatives, natural compounds, or ebsulfur/ebselen derivatives, have been nonpeptidomimetic.

**Figure 15:**
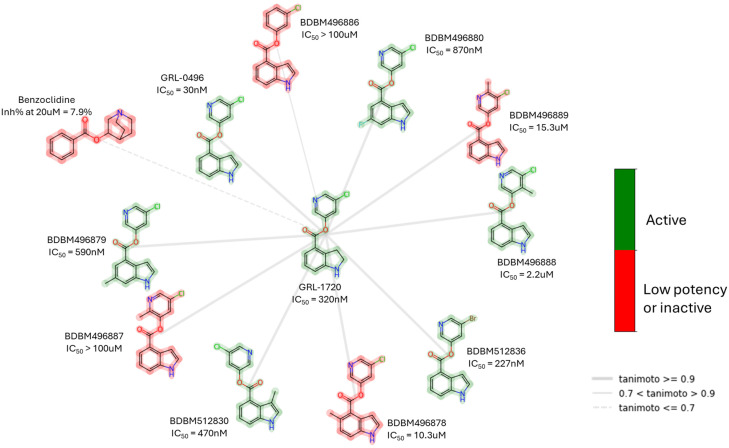
Small, irreversible covalent inhibitor of SARS-CoV-2 Mpro GRL-1720 and examples of its structurally closely related compounds (by maximum common substructure (MCS)) with known Mpro inhibitory activity from the PubChem Database. The low potency criterion was IC_50_ or *K*_i_ > 10 μM or Inh% at 20 μM < 75%.

In contrast to the peptidomimetic inhibitors, small-molecule Mpro covalent inhibitors display various binding modes and capacity to generate reversible or irreversible adducts. However, similar to peptidyl inhibitors, small-molecule Mpro inhibitors, according to the nature of the electrophilic warheads, are divided into the same groups: aldehydes, ketones, α-ketoamides, Michael acceptors, α-haloacetamides, nitriles, esters, and molecules containing an electrophilic selenium/sulfur atom. Among them, one of the significant groups is indole chlorpyridinyl esters, represented by the compound GRL-1720 (Figure 15).

### 3.4. Classification of Mpro Inhibitors by Electrophilic Warheads

In the context of inhibitors targeting the SARS-CoV-2 Mpro, electrophilic warheads are chemical groups designed to react with a specific amino acid residue (typically cysteine) in the enzyme’s active site. This reaction forms a covalent bond, effectively inhibiting the enzyme’s activity. It is important to note that, while covalent inhibitors can offer advantages such as increased potency and prolonged duration of action, they also present challenges such as off-target effects and potential toxicity. Careful design and optimization of these inhibitors are necessary to achieve selectivity and safety. Additionally, understanding the structure–activity relationship (SAR) of electrophilic warheads and their interactions with the enzyme’s active site is crucial for rationalizing effective Mpro inhibitors.

Aldehydes and Ketones. Depending on the specific chemical structure, these electrophilic groups can react reversibly or irreversibly with cysteine residues. Examples are compounds containing aldehyde or ketone functionalities, a benzotriazole-based compound.

α-Ketonamide. The α-ketoamide warhead is strategically chosen for its ability to form a stable covalent bond with the catalytic cysteine residue of Mpro. This irreversible inhibition mechanism ensures prolonged inhibition of enzyme activity, leading to potent antiviral effects. Examples are GC376 and the anti-HCV drugs boceprevir and telaprevir.

Michael Acceptors. These compounds contain an α,β-unsaturated carbonyl group that can undergo a Michael addition reaction with the thiol group of cysteine residues. Examples are α,β-unsaturated ketones, such as Michael acceptor inhibitors like peptidomimetic α-ketoamides (e.g., PF-07321332).

Nitriles. Nitrile-containing compounds can form a covalent bond with cysteine residues through a nucleophilic addition reaction. Examples are α-ketoamide nitrile inhibitors containing a nitrile warhead and a ketoamide moiety.

Others. Various other electrophilic functional groups, including chloroacetamides, chloromethyl ketones, ethynyl, and vinyl sulfones, may be employed as warheads in Mpro inhibitors. Table 2 summarizes the classification of Mpro inhibitors based on their electrophilic warheads.

## 4. Clinical Trials of SARS-CoV-2 Mpro Inhibitors

The focus of this section is to provide an extensive overview of clinical trials involving Mpro inhibitors. Specifically, we discuss examples of trials that have already been completed or are currently ongoing, with the compounds advancing to at least Phase 2 clinical trials. All clinical trial details are summarized in Table 3.

### 4.1. Covalent Inhibitors

Following the 2003 SARS outbreak, PF-00835231 emerged as an effective inhibitor against SARS-CoV-1 Mpro. Owen and his team introduced PF-07321332 (nirmatrelvir) in 2021 to enhance the limited absorption and permeability of PF-00835231 in animals, explicitly targeting SARS-CoV-2 Mpro [37]. This compound demonstrated potent antiviral activity in Vero E6 cells, with EC_50_ values of 0.074 μM, alongside remarkable selectivity and safety in vivo. To mitigate rapid metabolism of PF-07321332 by CYP3A, it was combined with the HIV protease inhibitor ritonavir, creating Paxlovid. Authorized by the United States Food and Drug Administration (FDA) on 22 December 2021, Paxlovid is indicated for treating adults with mild-to-moderate COVID-19 symptoms.

The outcome of FDA approval followed extensive clinical trials. Initially, a randomized, double-blind, placebo-controlled Phase 1 clinical trial was conducted to evaluate the safety, tolerability, and pharmacokinetics of nirmatrelvir in combination with ritonavir, used as a pharmacokinetic enhancer. The study included 70 healthy participants and evaluated single and multiple ascending doses of nirmatrelvir/ritonavir administered twice daily over 10 days in five parallel cohorts (NCT04756531) [93]. Subsequently, a double-blind, randomized, controlled Phase 2/3 study (NCT04960202) evaluated the efficacy and safety of nirmatrelvir in over 2200 adults, including symptomatic, unvaccinated, and non-hospitalized patients at high risk of progression to severe coronavirus infection. Participants were divided into two groups: one received 300 mg of nirmatrelvir and 100 mg of ritonavir, and the other received a placebo every 12 h for 5 days. Clinical results showed an 89% reduction in the risk of death or hospitalization related to COVID-19 in a cohort of adults treated with nirmatrelvir compared with those who received placebo.

Additionally, the nirmatrelvir cohort showed significantly lower viral loads and an acceptable safety profile [94]. In the following years, 2021–2023, Phase 1 clinical trials were also conducted on the impact of various Paxlovid formulations and high-fat meals on the drug’s bioavailability (NCT05129475, NCT05263895, NCT05339334). In addition, a safety study on Paxlovid was conducted among a cohort of eight healthy women who breastfed for a minimum of 12 weeks postpartum (NCT05441215), although the results have not yet been made public.

**Figure 16:**
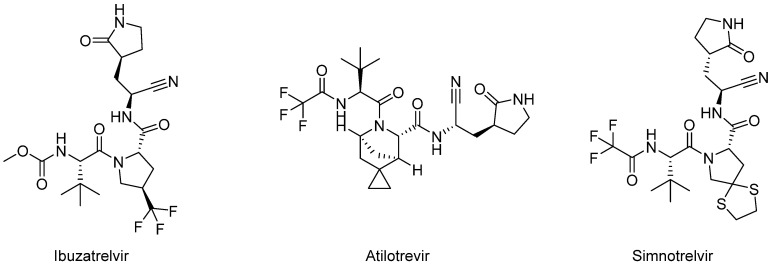
Structures of the second generation of Mpro inhibitors.

In its search for therapeutics against COVID-19, Pfizer has also conducted other clinical trials evaluating small-molecule therapeutics, including nirmatrelvir analogues such as PF-07304814 (lufotrelvir, Figure 10) and PF-07817883 (ibuzatrelvir, Figure 16).

Lufotrelvir, a phosphate prodrug, contains the active parent molecule PF-00835231, which has undergone Phase 1 clinical trials. Specifically, a double-blind, placebo-controlled Phase 1 study was conducted to evaluate the safety, tolerability, and pharmacokinetics of lufotrelvir at escalating single and multiple doses. Each study included two cohorts of eight adult patients, healthy participants (NCT04627532), or hospitalized patients with confirmed SARS-CoV-2 infection (NCT04535167). In these studies, no serious adverse events were reported with lufotrelvir, and it was noted that concentrations of the prodrug-derived active molecule PF-00835231 increased rapidly in a dose-dependent manner following intravenous infusions [95,96]. A subsequent Phase 1 clinical trial (NCT05050682) in five adult participants focused on characterizing mass balance and elucidating the drug’s pharmacokinetics, metabolism and excretion profiles as a continuous intravenous infusion at a dose of 500 mg over 24 h.

Another nirmatrelvir analogue, PF-07817883 (ibuzatrelvir, Figure 16), is a second-generation oral inhibitor of SARS-CoV-2 Mpro developed by Pfizer. Its design is derived from the structural framework of nirmatrelvir. PF-07817883 exhibits extensive antiviral activity against coronaviruses in vitro and demonstrates efficacy in a mouse-adapted SARS-CoV-2 model following oral administration. The enhanced metabolic stability of PF-07817883, compared to nirmatrelvir, potentially eliminates the requirement for coadministration with a cytochrome P450 inhibitor. Additionally, in vitro assessments indicate that ibuzatrelvir has low potential for drug-drug interactions [97]. A randomized, double-blind, placebo-controlled Phase 1 clinical trial (NCT05580003) was designed to study drug pharmacokinetics and drug-drug interactions in 94 healthy adults. Furthermore, an additional Phase 1 clinical trial was conducted (NCT06122194) to evaluate novel drug formulations relative to a reference drug. This study aimed to compare the pharmacokinetic profiles and assess the safety and efficacy of these new formulations. A recent Phase 2 clinical trial assessed the safety and effectiveness of ibuzatrelvir in over 200 adult participants with COVID-19 symptoms who did not need to be hospitalized (NCT05799495). The results of the aforementioned clinical trials have not yet been published.

Atilotrevir (GST-HG171, Figure 16) is a highly effective Mpro inhibitor taken orally and has shown superior potency and effectiveness in preclinical trials compared to nirmatrelvir. Consequently, the drug underwent a clinical trial to assess its efficacy, safety, tolerability, and pharmacokinetics when administered in combination with ritonavir (NCT05668897). This trial followed a randomized, double-blind, placebo-controlled design and involved 78 healthy participants. The effects of atilotrevir were investigated by administering doses of 150 and 300 mg, either twice or three times daily, over 5 days. These studies were conducted without any occurrence of serious adverse events. Due to its favorable safety and efficacy profile, the dosage combination of 150 mg atilotrevir/100 mg ritonavir taken twice daily was selected for a further Phase 2 clinical trial (NCT05656443) [98]. This Phase 2, multicenter, randomized, double-blind, placebo-controlled clinical trial evaluated the efficacy and safety of coadministration of atilotrevir and ritonavir for treating mild-to-moderate COVID-19 in over 1200 participants. Most participants in the Phase 2 clinical trial (over 95%) had completed basic or booster vaccination against COVID-19 and were at low risk of disease progression at baseline. According to the study, coadministration of atilotrevir and ritonavir leads to a notable reduction in the duration of symptoms and clearance of the virus in low-risk (vaccinated) COVID-19-infected adult patients. Adverse events occurred with similar frequency in the study and placebo groups, and the most common was hypertriglyceridemia [99]. A single-center, non-randomized, open-label, parallel Phase 1 clinical trial with a single dose of atilotrevir was also recently initiated to evaluate safety and pharmacokinetics in patients with hepatic impairment (NCT06106113). Additionally, a separate, completed, open-label Phase 1 study examined the effect of a potent CYP3A4 inhibitor, itraconazole, on the pharmacokinetics of atilotrevir/ritonavir in 12 healthy participants (NCT06087055).

Simnotrelvir (SIM-0417, Figure 16) is another selective Mpro inhibitor that can effectively combat SARS-CoV-2 infection. The safety, tolerability, and pharmacokinetics of simnotrelvir administered as monotherapy and in combination with ritonavir were assessed in a Phase 1 clinical trial in over 100 healthy participants (NCT05339646). Furthermore, a clinical trial was carried out during Phase 1b/2 to investigate the safety, pharmacodynamics, and pharmacokinetics of simnotrelvir/ritonavir in 32 adult participants with asymptomatic and mild COVID-19. The trial followed a randomized, double-blind, and placebo-controlled design and administered simnotrelvir/ritonavir at two different doses: 750/100 mg and 300/100 mg (NCT05369676). Results demonstrated that simnotrelvir/ritonavir is a generally safe and well-tolerated combination at a dose of 750 mg/100 mg twice daily under fasted conditions, and this dose was selected for further study [100,101]. Simnotrelvir/ritonavir was subsequently enrolled in a multicenter, randomized, double-blind Phase 2/3 clinical trial to evaluate efficacy and safety in over 1200 adult patients with symptomatic and mild to moderate COVID-19 disease (NCT05506176). During this study, participants were administered a dosage of 750 mg of simnotrelvir combined with 100 mg of ritonavir or a placebo twice daily for 5 days. This treatment was initiated within a 72-h window from the onset of symptoms. Participants in the simnotrelvir group had a significantly shorter time to permanent resolution of COVID-19 symptoms and a lower viral load than those in the placebo group. Mild to moderate side effects were noticed during the study [102]. Additionally, open-label Phase 1 studies have been completed to evaluate the drug-drug interactions of itraconazole, rifampicin, and midazolam with simnotrelvir/ritonavir in more than 30 Chinese participants (NCT05665647). A separate Phase 1 study assessed mass balance, biotransformation, safety, and tolerability after a single oral dose of simnoltrevir in combination with ritonavir in six healthy adult Chinese men (NCT05475834). The results of these studies have not yet been published.

Additionally, multiple covalent inhibitors coadministered with ritonavir have recently completed Phase 1 safety and pharmacokinetics studies in healthy volunteers, including EDP-235 (NCT05246878), QLS1128 (NCT05458076), and FB2001 (bofutrelvir, NCT04766931, NCT05197179), and are currently recruiting for Phase 2 clinical trials (NCT05445934, NCT05616728, NCT05689203).

**Figure 17:**
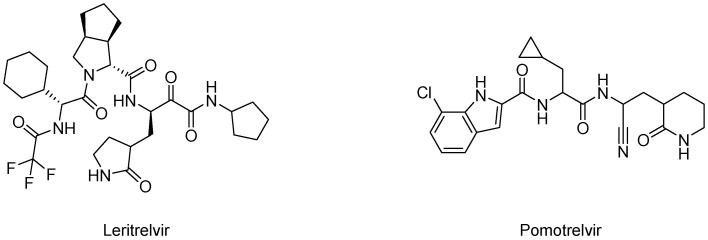
Structures of leritrelvir and polmotrelvir.

Another example of an Mpro inhibitor is the novel α-ketoamide-based molecule leritrelvir (RAY1216, Figure 17), whose safety, tolerability, pharmacokinetics, and the influence of food intake on its effectiveness were evaluated in double-blind, randomized, single, and multiple-dose Phase 1 trial on 88 healthy adult participants (NCT05829551). Subsequently, its interactions with midazolam, omeprazole, rosuvastatin, verapamil, and rifampicin were examined in an open-label, randomized, Phase 1 study of 56 adult participants (NCT06031454). Then, a Phase 2 clinical trial demonstrated that leritrelvir, with or without coadministration of ritonavir, demonstrated comparable antiviral efficacy and safety [103]. In another randomized, double-blind, placebo-controlled, multicenter Phase 3 clinical trial (NCT05620160) conducted in China, the efficacy of leritrelvir monotherapy was evaluated in over 1300 participants with mild-to-moderate symptoms of COVID-19. The study lasted up to 28 days, and participants received 400 mg of leritrelvir thrice daily for five days. The results showed that leritrelvir monotherapy effectively treated mild-to-moderate COVID-19, without raising major safety concerns. Hypertriglyceridemia and hyperlipidemia were the most frequently reported adverse events, but none of them were considered severe. Additionally, leritrelvir has recently been studied in various single-dose, non-randomized, open-label Phase 1 clinical trials to assess changes in its pharmacokinetics in patients with severe renal disease (NCT06160622), patients with hepatic impairment (NCT06161259), and healthy elderly subjects (NCT06169085).

Pomotrelvir (PBI-0451, Figure 17) is a potent, competitive inhibitor of SARS-CoV-2, demonstrating antiviral activity in both enzymatic and cellular assays, with high selectivity for human receptors and proteases in vitro. Toxicological studies, both in vitro and in vivo, did not reveal any significant adverse effects. Based on these results, a Phase 1 study was initiated, consisting of a placebo-controlled, blinded, randomized, dose-escalation, drug-interaction study in 130 healthy participants (NCT05011812). Subsequently, a double-blind, randomized, Phase 2 study of pomotrelvir in 210 non-hospitalized adults with symptomatic COVID-19 assessed its antiviral activity, safety, and efficacy after oral administration (700 mg (2 × 350 mg tablets) twice daily) compared to placebo (NCT05543707). The tested dose reached and maintained concentrations in this clinical trial phase, ensuring strong antiviral activity against SARS-CoV-2.

### 4.2. Non-Covalent Inhibitors

Besides Paxlovid, another molecule called ensitrelvir (S-217622, Figure 5) was approved in Japan on 22 November 2022. The pharmaceutical company Shionogi developed the drug in cooperation with Hokkaido University. The molecule was discovered through virtual screening, followed by biological assay and optimization of the hit compound using a structure-based drug design approach. The optimization process involved enhancing the P1′ ligand based on a hit compound to compound, where the P1′ ligand was transformed into 6-chloro-2-methyl-2H-indayzole, boosting its enzyme inhibitory activity by 90-fold. Subsequently, the methylamide moiety of P1 was replaced by various heterocyclic compounds to generate ensitrelvir. This compound displayed significant inhibitory activity against SARS-CoV-2 Mpro as a non-covalent and nonpeptidic inhibitor, with an IC_50_ value of 0.013 μM and effective antiviral activity, with an EC_50_ value of 0.37 μM. It exhibited antiviral activity against a range of SARS-CoV-2 variants and coronaviruses in vitro, along with favorable drug metabolism and pharmacokinetic profiles for oral dosing. These included high metabolic stability (96% and 88% in human and rat liver microsomes, respectively), high oral absorption (97%), and low clearance (1.70 mL/min/kg) in rats [104].

Given the promising preclinical results, ensitrelvir was included in clinical trials, which are still ongoing. A multicenter, randomized, double-blind, placebo-controlled Phase 1 study (jRCT2031210202) was conducted to evaluate the safety and pharmacokinetics of ensitrelvir tablets administered once daily to healthy volunteers, both Japanese and white women and men. The Mpro inhibitor was well tolerated, and no major safety concerns were identified. The pharmacokinetics of ensitrelvir were similar in all study populations [105]. In Phase 2/3 clinical trials, over 300 participants were orally administered the Mpro inhibitor ensitrelvir (jRCT2031210350). The study results showed that the viral load decreased, but the symptoms of infection did not significantly improve in both Japanese and white women and men. The Mpro inhibitor was well tolerated, and no major safety concerns were identified [105]. Additionally, ensitrelvir was associated with an almost 40% reduction in the loss of smell and taste after SAR-CoV-2 infection [106]. The most common side effects include a transient decrease in high-density lipoprotein levels and increased triglycerides in the blood. Based on the result, ensitrelvir received Japan’s first emergency and full approval. Multiple ongoing Phase 2 or 3 clinical trials (NCT05897541, NCT06161688, NCT05605093, NCT05305547) investigate ensitrelvir. These studies are designed to evaluate the safety and effectiveness of ensitrelvir in several contexts: preventing symptomatic SARS-CoV-2 infection among household contacts of people infected with COVID-19, treating adults with long-term COVID-19 infection, or improving outcomes in hospitalized patients with COVID-19 and effectiveness in adult outpatients with mild-to-moderate symptoms of COVID-19 initiating treatment within three days of symptom onset.

**Figure 18:**
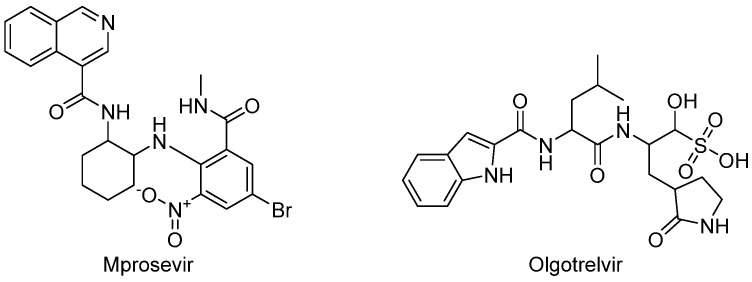
Structures of mprosevir and olgotrelvir.

Like ensitrelvir, mprosevir (WU-04, WPV01, Figure 18) acts as a non-covalent Mpro inhibitor, effectively impeding SARS-CoV-2 replication in human cells at nanomolar concentrations. Ensitrelvir seems metabolically stable, so it is unnecessary to coadminister it with ritonavir. This molecule core is based on isoqunoline, which was discovered by a research team at West Lake University through DNA-encoded library technology screening [107]. Preclinical studies indicate that mprosevir has anti-SARS-CoV-2 activity comparable to nirmatrelvir in K18-hACE2 mice when administered orally at equivalent doses [108,109]. After preclinical studies, mprosevir entered Phase 1 clinical trials (NCT06205329), which examined the drug’s safety, tolerability, and pharmacokinetics alone and coadministered with ritonavir in 108 healthy adult patients. A randomized, double-blind, placebo-controlled Phase 2 clinical trial (NCT05752175) was developed with 80 participants with mild/moderate COVID-19, which assessed the effectiveness and safety of mprosevir administrated without ritonavir, but the results of this study are not yet available. Mprosevir is being evaluated in a multicenter, randomized, double-blind, placebo-controlled Phase 3 clinical trial (NCT06197217) in over 1300 patients with mild-to-moderate COVID-19 disease to test its efficacy and safety.

### 4.3. Dual Inhibitor of SARS-CoV-2 Mpro and Cathepsin L

Olgotrelvir is another drug candidate against the SARS-CoV-2 virus. This compound stands out from others because of its dual mechanism. The primary purpose of designing olgotrelvir was to inhibit SARS-CoV-2 Mpro covalently; however, it has been found that the compound may have an additional mechanism that could potentially increase its antiviral effectiveness. This additional mechanism is based on inhibition of the activity of human lysosomal cysteine protease cathepsin L (CTSL), which prevents the virus from entering the endosome. The compound’s dual mechanism is expected to result in enhanced antiviral activity and lower the risk of drug resistance development in the virus.

A Phase 1 study has been conducted to evaluate the pharmacokinetics and safety of olgotrelvir (NCT05364840, Figure 18) [110]. It was a two-part, randomized, double-blind, placebo-controlled, single and multiple ascending dose study, which 58 healthy volunteers attended. Subjects received a single ascending dose of 2000 mg or a multiple ascending dose of 800 mg twice daily for 7.5 days. Toxicity was not detected in the study. Next, a separate Phase 1 study was conducted to explore the antiviral activity of olgotrelvir in COVID-19 patients (NCT05523739). Forty-one patients with asymptomatic or mild COVID-19 were included in this study. They were treated with a 300 mg, 600 mg, or 800 mg dose twice daily for 7.5 days. As the results of this study, it was shown that, in each group, there was a reduction of SARS-CoV-2 viral RNA copies. After that, a Phase 3 trial was conducted (NCT05716425) [111]. In total, 1212 mild or moderate COVID-19 patients participated in this study. All patients were treated with 600 mg of olgoterlvir twice daily for 5 days or with a placebo. The result was a reduction in recovery time by 2.4 days, as evidenced by the resolution of 11 COVID-19-related symptoms. In addition, it has been shown that the viral RNA copy load was reduced on day 4 of treatment.

In conclusion, olgotrelvir is a promising candidate as a new drug against the SARS-CoV-2 virus. The argument for this is its dual mechanisms, which may increase activity and reduce viral resistance compared to other candidates, but also the positive results of the clinical trials presented above.

## 5. Conclusions, Challenges, and Future Directions

Based on the clinical studies described above, nirmatrelvir has emerged as the most effective Mpro inhibitor to date, as its discovery in March 2020 led to Emergency Use Authorization in December 2021, followed by full FDA approval in March 2023. This accelerated development was supported by prior work on the Mpro inhibitor PF-00835231, initiated in response to the 2002 SARS epidemic. Development of PF-00835231 was halted due to its poor oral bioavailability and the disappearance of SARS-CoV in late 2004. PF-00835231 as an early lead was optimized to nirmatrelvir by adding a nitrile head, a γ-lactam in the P1 region, and a bicyclic proline derivative in the P2 region [9,37,87]. Moreover, nirmatrelvir is primarily metabolized by the human hepatic enzyme cytochrome P450 3A4 (CYP3A4). Therefore, to increase its therapeutic concentration and effectively inhibit SARS-CoV-2 replication, it should be coadministered with a strong CYP3A4 inhibitor, such as ritonavir. However, preliminary studies have shown sporadic cases of disease recurrence during SARS-CoV-2 infection after treatment with Paxlovid [9,112]. Hence, other Mpro inhibitors similar in terms of antiviral activity but with a different molecular structure, such as ibuzatrelvir, atilotrelvir, and simnotrelvir, in combination with ritonavir have entered clinical trials but have also demonstrated high susceptibility to CYP3A4 metabolism. The next molecules in clinical trials, leritrelvir and pomotrelvir, showed increased stability compared to their predecessors without the need to administer them with the CYP P450 inhibitor ritonavir.

Effective Mpro inhibitors represent key tools in the treatment of COVID-19, potentially reducing the risk of transmission, disease severity, and mortality, particularly among patients with multiple comorbidities. Therefore, in the subsequent phases of clinical trials, after confirming the effectiveness of the Mpro inhibitor, studies are conducted to assess the possibility of administering these drugs to patients with liver or kidney failure, older adults, or breastfeeding women. Such studies have shown that Paxlovid may interact with CYP3A4 inducers or pose a risk in patients with severe renal or hepatic impairment [113]. Similar clinical trials to evaluate potency, safety, and drug–drug interactions have also been conducted for other Mpro inhibitors such as symnotrelvir, atilotrelvir, and letrelvir.

Clinical trials of Mpro inhibitors have included compounds acting as non-covalent inhibitors of this protein, such as entirelvir and mprosevir. Unlike covalent inhibitors such as nirmatrelvir, which form a covalent bond to a nucleophilic site in a protein via a reactive functional group, non-covalent inhibitors interact with their targets via weaker forces such as hydrogen bonds, van der Waals interactions, and hydrophobic interactions. Although covalent inhibitors can be very effective, they may also be associated with a greater risk of side effects and toxicity. In contrast, non-covalent inhibitors usually have greater selectivity and often cause fewer side effects [114]. In the case of the results of clinical trials of both types of inhibitors, the time to resolution of COVID-19 symptoms and the extent of viral replication were reduced. An advantage of clinical trials involving enzitrevir, a non-covalent Mpro inhibitor, is that they enroll patients regardless of their risk profile for severe disease, unlike trials focused on nirmatrevir. Additionally, unlike other studies that primarily enrolled unvaccinated patients receiving oral antivirals, this study included both vaccinated and unvaccinated individuals who were infected [115]. Non-covalent inhibitors are typically preferred in antiviral therapy due to their favorable safety profile, more predictable pharmacokinetic properties, and fewer side effects. However, designing effective antiviral compounds that act through this mechanism is challenging [114], resulting in fewer non-covalent inhibitors currently in clinical trials for Mpro inhibitors. Nevertheless, both types of inhibitors play a key role in combating a wide range of viral diseases.

Mpro plays a key role in the replication of coronaviruses, making it a prime target for antiviral drugs. It is particularly promising for anti-coronavirus therapies due to its widespread conservation among strains, specific cleavage sequence preferences, and lack of similar proteases in host cells. Natural mutations, such as E166V, found in the Mpro protein may potentially cause resistance to the approved drugs nirmatrelvir and enzitrelvir [9,14]; hence the need to search for compounds with a broader or multidirectional mechanism of action.

An example of a dual-acting compound in clinical trials is olgotrelvir, an oral antiviral compound that targets both Mpro and human cathepsin L. Combining these two mechanisms of action may increase effectiveness against different virus variants and reduce the emergence of resistance. This approach may lead to more potent viral suppression, faster resolution of symptoms, and potentially a reduced incidence of severe disease or complications. By targeting multiple pathways, dual inhibitors may pose a more significant challenge to viral mutation and resistance development than single-target therapies such as nirmatrelvir [116]. Overall, dual Mpro inhibitors represent a promising approach in the fight against COVID-19, leveraging multiple mechanisms to combat the virus effectively.

Summing up, developing antiviral drugs requires extensive clinical trials to ensure efficacy, safety, and specificity, and both covalent and non-covalent inhibitors continue to be an essential area of interest in drug development. Moreover, successful trials of Mpro inhibitors can pave the way for developing other antiviral drugs targeting similar proteases in different viruses. On the other hand, many clinical trials with Mpro inhibitors were discontinued after Phase 1 because of inadequate pharmacokinetic profiles or poor bioavailability despite promising results from in vitro or animal models.

During and after the pandemic, the ability to rapidly develop and test new compounds in preclinical and clinical trials has significantly improved, increasing preparedness for future viral epidemics. However, the urgent need for new antiviral drugs must not disturb the balance between new treatments and the rigorous standards required in clinical trials. This is particularly important considering that clinical trials are expensive and time-consuming and require significant financial, time, and human resource investment.

Our analysis of recent reports on developing SARS-CoV-2 Mpro inhibitors demonstrates significant advancements in their potential as therapeutic drugs against SARS-CoV-2. Noteworthy progress has been made in clinical trials of nirmatrelvir and ensitrelvir. The fact that these inhibitors effectively reduce viral load and improve patient outcomes emphasizes the viability of Mpro as a target for antiviral therapy. However, the widespread use of inhibitors like nirmatrelvir and ensitrelvir is likely to drive the selection of resistant strains of SARS-CoV-2. This resistance could reduce the long-term efficacy of these treatments, necessitating the continuous development of novel inhibitors or combination therapies to stay ahead of viral mutations. In order to ensure the maximum effectiveness and safety of new inhibitors, researchers need to acknowledge and address the numerous challenges that arise during the transition from hit discovery to clinical studies.

The unfavorable properties of drug metabolism and pharmacokinetics (PK) pose a significant barrier to further developing new Mpro inhibitors. The effectiveness of many developed Mpro inhibitors as orally administered drugs is limited due to their poor oral bioavailability, rapid clearance, and extensive metabolism. For instance, despite its potency, lufotrelvir has poor oral bioavailability, necessitating intravenous administration, which is less convenient and limits its use. Toxicity poses another major obstacle. The progress of specific Mpro inhibitors, including walrycin B or shikonin, through preclinical trials, was impeded as they demonstrated cytotoxicity towards Vero E6 cells used in biological tests.

To overcome these challenges, future research should focus on several key areas. To effectively address the challenges of SARS-CoV-2, it is crucial to move away from using scaffolds derived from inhibitors of SARS-CoV-1 Mpro. The exploration of new chemical spaces and scaffold-hopping has the potential to yield inhibitors that are more effective and safer. The promising direction indicated by olgotrelvir, which combines Mpro inhibitory effects with another mechanism to improve effectiveness, should be further explored. Dual inhibitors can simultaneously target multiple pathways involved in viral replication or host–virus interaction, which could lower the chances of resistance and improve the effectiveness of antiviral treatment. Combining Mpro inhibitors with other antiviral agents or immune modulators can improve therapeutic outcomes, as this provides a comprehensive approach to fighting the virus, minimizing the likelihood of resistance and promoting better patient outcomes. Some potential biological targets include the inhibition of host proteases like cathepsin L, the targeting of viral RNA-dependent RNA polymerase to impede viral genome replication, and the modulation of host immune responses to strengthen antiviral defense and minimize the harmful effects of inflammation caused by COVID-19. To ensure the successful development of Mpro inhibitors, it is vital to consider PK properties from the early stages, which involves enhancing oral bioavailability, optimizing metabolic stability, and reducing toxicity. Medicinal chemistry efforts should focus on modifying molecular structures to achieve these goals without compromising potency.

In conclusion, while the development of Mpro inhibitors has made commendable progress, addressing the challenges of PK properties, toxicity, and resistance is critical. Future research should focus on novel scaffolds, dual inhibitors, and combination therapies with molecules targeting additional biological mechanisms to enhance the efficacy and safety of these promising antiviral agents.

## Figures and Tables

**Table 1:** Advantages and disadvantages of peptidomimetics and small molecules in drug discovery.

Peptidomimetics	Small Molecules
Advantages	
Mimic the complex interactions of natural peptides with their receptors or other targets.	Can interact with a wide range of biological targets. Versatile tools in drug discovery
Like natural peptides, showed high specificity and selectivity for targets, fewer off-target effects, and reduced toxicity.	Better membrane permeability, easily cross the cell membranes, reaching intracellular targets.
Improved metabolic stability compared to natural peptides	Good oral bioavailability, more convenient for patients.
Disadvantages	
Many peptidomimetics still suffer from poor oral bioavailability and may require parenteral administration.	Small molecules can be rapidly metabolized and excreted, sometimes requiring frequent dosing or leading to short half-lives.
Compared to small molecules, inadequate membrane permeability	High potential for off-target effects and toxicity.
Complex and expensive synthetic processes are barriers to large-scale production.	Limited capability to mimic complex and specific interactions of larger biomolecules like peptides.
They can sometimes elicit immune responses.	More prone to the development of drug resistance.

**Table 2:** Classification of Mpro inhibitors based on their electrophilic warheads.

Warhead	Mechanism of Action	Example
Carbonyl 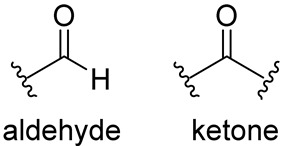	The nucleophilic addition of the cysteine -SH leads to the formation of a reversible hemithioacetal adduct. 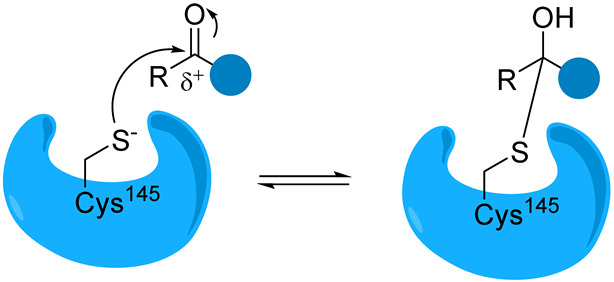	(a) aldehyde GC373 GC376 [86] NK01-63 (*coronastat*) (b) ketone PF-00835231 PF-07304814 [87]
α-Ketonamide 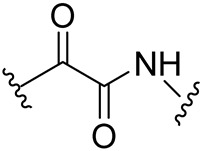	The α-ketoamide moiety forms additional non-covalent H bonds with the amino acids of the active site via the carbonyl oxygen and the -OH of the hemithioacetal 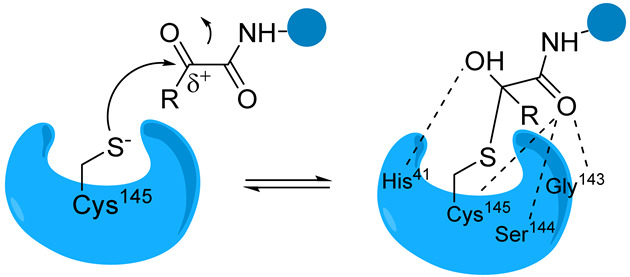	Boceprevir [76] Telaprevir [88] UAWJ246 UAWJ248 [20]
Michael acceptor group 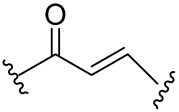	Michael acceptor groups inhibit the enzymes via conjugate addition of the nucleophilic cysteinyl -SH to the electrophilic C*β* of the unsaturated system, producing a nearly irreversible and longer-lasting adduct. 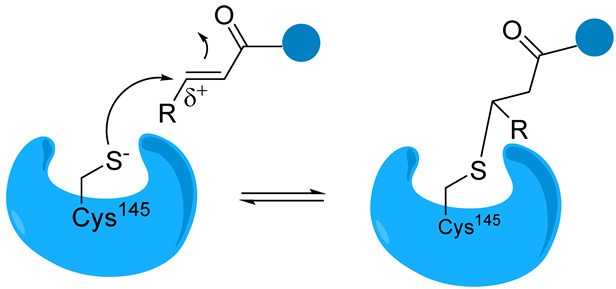	Compound N3 [89,90] SIMR-2418 [34] Cinanserin [32] ML188 [91]
Nitrile 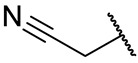	Due to the difference in electronegativity with the nitrogen atom, the nitrile carbon is vulnerable to nucleophilic addition by the protease’s catalytic cysteine, leading to the formation of a reversible thioimidate covalent adduct. 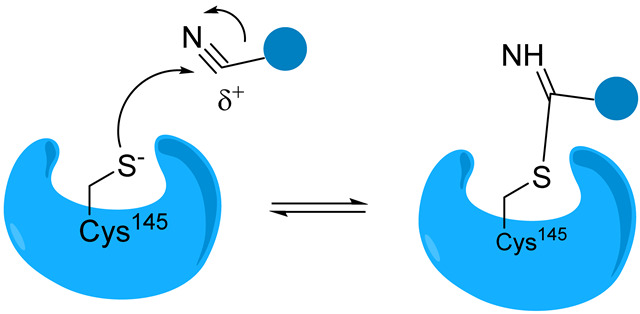	PF-07321332 (*nirmatrelvir*) [37] PF-07817883 [92] GST-HG171 (*atilotrevir*) [36]

**Table 3:** Clinical trials of selected Mpro inhibitors.

Active Compound (Sponsor)	ClinicalTrials.gov Identifier	Phase	Summary of the Study
Nilmatrelvir (PF-07321332)/ Ritonavir (Pfizer, New York, NY, USA)	NCT04756531	1	Study of PF-07321332 in healthy participants.
	NCT04960202	2/3	EPIC-HR: Study of Oral PF-07321332/Ritonavir Compared with Placebo in Nonhospitalized High Risk Adults With COVID-19.
	NCT05047601	2/3	A Study of a Potential Oral Treatment to Prevent COVID-19 in Adults Who Are Exposed to Household Member(s) With a Confirmed Symptomatic COVID-19 Infection.
	NCT05129475	1	Food Effect Study to Evaluate the Effect of High-Fat Meal on the Relative Bioavailability of PF-07321332 Boosted with Ritonavir in Healthy Adult Participants.
	NCT05263895	1	Relative Bioavailability Study of 4 Different Formulations of PF-07321332 Relative to the Commercial Tablet Formulation.
	NCT05339334	1	A Study to Learn About the Study Medicine PF-07321332 and Ritonavir in Adult Healthy Chinese Participants.
	NCT05441215	1	A Study to Learn About the Medicine (PF-07321332 or Nirmatrelvir/Ritonavir) in Healthy Lactating Women.
	NCT05263921	1	Relative Bioavailability Study of PF-07321332/Ritonavir Oral Powder Relative to the Commercial Tablets in Healthy Participants.
Lufotrelvir (PF-07304814)/ Ritonavir (Pfizer, New York, NY, USA)	NCT04535167	1	First-In-Human Study to Evaluate Safety, Tolerability, And Pharmacokinetics Following Single Ascending and Multiple Ascending Doses of PF-07304814 In Hospitalized Participants With COVID-19.
	NCT04627532	1	Single Ascending Dose Study of Intravenous Infusion of PF 07304814 in Healthy Adult Participants.
	NCT05050682	1	Study to Investigate the Mass Balance, Metabolism, and Excretion of [^14^C]-PF-07304814 in Healthy Participants.
Ibuzatrelvir (PF- 07817883)/ Ritonavir (Pfizer, New York, NY, USA)	NCT05580003	1	A Study to Learn Safety and Blood Levels of PF-07817883 in Healthy People.
	NCT05799495	2	A Study to Understand the Effect and Safety of the Study Medicine PF-07817883 in Adults Who Have Symptoms of COVID-19 But Are Not Hospitalized.
	NCT06122194	1	A Study to Learn PF-07817883 Blood Levels After Administration of Tablets of Study Drug to Healthy Adult Volunteers.
Atilotrelvir (GST-HG171)/ Ritonavir (Fujian Akeylink Biotechnology Co., Ltd., Ningde, Fujian, China)	NCT05668897	1	Safety, Tolerability and Pharmacokinetic Characteristics Evaluation on GST-HG171 Tablets.
	NCT05656443	2/3	Study of GST-HG171/Ritonavir Compared with Placebo in Patients with Mild to Moderate COVID-19.
	NCT06106113	1	Pharmacokinetics and Safety of GST-HG171 Tablets in Subjects with Impaired and Normal Liver Function.
	NCT06084507	1	Food Effects of GST-HG171 Tablets Combined with Ritonavir in Healthy Chinese Participants.
	NCT06087055	1	Drug-Drug Interaction Study of Itraconazole With GST-HG171/Ritonavir in Healthy Participants.
Simnotrelvir (SIM-0417, SSD8432)/ Ritonavir (Jiangsu Simcere Pharmaceutical Co., Ltd., Nanjing, China)	NCT05339646	1	A Phase 1 Clinical Study of SSD8432 in Healthy Adult Subjects.
	NCT05369676	1/2	To Evaluate SSD8432/Ritonavir in Adults With COVID-19.
	NCT05475834	1	Study to Investigate the Mass Balance and Biotransformation of SIM0417 in Healthy Adult Chinese Male Participants.
	NCT05506176	2/3	A Clinical Study to Evaluate the Efficacy and Safety of SIM0417 Orally Co-Administered with Ritonavir in Symptomatic Adult Participants with Mild to Moderate COVID-19.
	NCT05665647	1	Drug-Drug Interaction Study of Itraconazole, Rifampicin and Midazolam with SIM0417/Ritonavir in Healthy Participants.
Leritrelwir (RAY1216) (Guangdong Raynovent Biotech Co., Ltd., Guangzhou, China)	NCT05829551	1	The Safety, Tolerability and Pharmacokinetics Study of RAY1216 in Healthy Adult Participants.
	NCT05620160	3	Study of RAY1216 Tablets Compared with Placebo in Patients with Mild to Moderate COVID-19.
	NCT06031454	1	Drug-durg Interaction of Leritrelvir (RAY1216) With Midazolam, Omeprazole, Rosuvastatin, Verapamil, and Rifampin.
	NCT06169085	1	Pharmacokinetics of Leritrelvir (Ray1216) in Elder Participants.
	NCT06160622	1	Pharmacokinetics of Leritrelvir (RAY1216) in Participants with Severe Kidney Disease.
	NCT06161259	1	Pharmacokinetics of Leritrelvir (RAY1216) in Participants with Hepatic Impairment.
	NCT06362460	1	Mass Balance Study of [^14^C] RAY1216 in Healthy Adult Male Subjects in China.
Pomotrelvir (PBI-0451) (Pardes Biosciences, Inc., Carlsbad, CA, USA)	NCT05011812	1	Study of PBI-0451 in Healthy Subjects.
	NCT05543707	2	PBI-0451 (Pomotrelvir) Phase 2 Study in Nonhospitalized Symptomatic Adults With COVID-19.
Ensitrelvir (S-217622)	jRCT2031210202 *	1	A Phase 1 study of S-217622.
(Nagata Tsute, Osaka, Japan)			
(Gomez JuanCarlos, Osaka, Japan)	jRCT2031210350 *	2/3	A Phase 2/3 study of S-217622.
(Timothy Henrich, San Francisco, CA, USA)	NCT06161688	2	Ensitrelvir for Viral Persistence and Inflammation in People Experiencing Long COVID (PREVAIL-LC).
(Shionogi Inc., Osaka, Japan)	NCT05897541	3	Phase 3 Study of S-217622 in Prevention of Symptomatic SARS-CoV-2 Infection (SCORPIO-PEP).
	NCT05305547	3	A Study to Compare S-217622 With Placebo in Non-Hospitalized Participants With COVID-19 (SCORPIO-HR).
(University of Minnesota, Minneapolis, MN, USA)	NCT05605093	3	Strategies and Treatments for Respiratory Infections & Viral Emergencies (STRIVE): Shionogi Protease Inhibitor (Ensitrelvir).
Mprosevir (WPV01, WU-04)/Ritonavir (Westlake Pharmaceuticals (Hangzhou) Co., Ltd., Hangzhou, China)	NCT06205329	1	Study of WPV01 in Healthy Subjects.
	NCT05752175	2	Study of WPV01 Compared with Placebo in Patients with Mild/Moderate COVID-19 Infection
	NCT06197217	3	Phase 3 Clinical Study Evaluating the Efficacy and Safety of WPV01 in Patients with Mild/Moderate COVID-19.
Olgotrelvir (STI-1558)	NCT05364840	1	Study to Assess the Safety, Tolerability and Pharmacokinetics of STI-1558 in Healthy Volunteers.
(Sorrento Therapeutics, Inc., San Diego, CA, USA)			
(Zhejiang ACEA Pharmaceutical Co., Ltd., Hangzhou, China)	NCT05523739	1	Safety and Efficacy Study of STI-1558 in Healthy Adults and SARS-CoV-2-Positive Patients.
(Zhejiang ACEA Pharmaceutical Co., Ltd., Hangzhou, China)	NCT05716425	3	Study to Assess the Efficacy and Safety of STI-1558 in Adult Subjects with Mild or Moderate.
(Zhejiang ACEA Pharmaceutical Co., Ltd., Hangzhou, China)	NCT05754411	1	Study on Mass Balance and Biotransformation of STI-1558 in Healthy Chinese Adult Male Subjects.
(Zhejiang ACEA Pharmaceutical Co., Ltd., Hangzhou, China)	NCT06044233	1	A Bioequivalence Trial of Fasting Single Oral STI-1558 Capsule in Healthy Chinese Subjects.

* Japan Primary Registries Network number.

## Data Availability

The raw data supporting the conclusions of this article will be made available by the authors on request.
